# Salidroside protects against high-altitude hypoxia-induced kidney injury via regulation of renal dopamine D1-like receptors

**DOI:** 10.1371/journal.pone.0344999

**Published:** 2026-03-31

**Authors:** Cheng Huan, Gan Zhilin, Xiao Dan, Wang Yue, Li Xianglian, Mo Liwen, Cheng Yue

**Affiliations:** 1 College of Medicine, Southwest Jiaotong University, Chengdu, PR China; 2 Chengdu Chengnan Jinhua Hospital, Chengdu, PR China,; 3 Department of Nephrology, General Hospital of Western Theater Command of PLA, Chengdu, PR China; Hirosaki University Graduate School of Medicine, JAPAN

## Abstract

High-altitude hypoxia is a well-established risk factor for acute kidney injury (AKI), yet effective therapeutic options remain scarce. Salidroside, the primary active compound extracted from Rhodiola, has been reported to protect against hypoxia-induced damage in various organs. Here, we aimed to determine whether salidroside could alleviate kidney injury caused by acute high-altitude exposure and to investigate its underlying mechanisms. To this end, male Sprague-Dawley rats were exposed to hypobaric hypoxia simulating an altitude of 5000 meters and were treated with different doses of salidroside. Kidney injury biomarkers, including neutrophil gelatinase-associated lipocalin (NGAL), kidney injury molecule-1 (KIM-1), and cystatin C (Cys-C), were measured in serum and urine. Histological analysis and protein expression levels of dopamine D1-like receptor (DRD1) and G protein-coupled receptor kinase 4 (GRK4) were also evaluated. In parallel, primary renal proximal tubular (RPT) cells from rats were cultured under hypoxic conditions to validate the findings in vitro, with additional groups receiving DRD1-targeting siRNA or the DRD1 agonist fenoldopam. Salidroside significantly reduced biomarker levels of kidney injury in vivo, preserved DRD1 expression, and inhibited GRK4 upregulation in a time- and dose-dependent manner. Likewise, in vitro treatment with salidroside enhanced cell viability and decreased apoptosis while restoring DRD1 levels and downregulating GRK4. Notably, the protective effects were abolished by DRD1 knockdown and enhanced by fenoldopam, indicating a DRD1-dependent mechanism. Molecular docking analysis further supported these results by demonstrating strong binding affinities between salidroside and both DRD1 and GRK4. Together, our findings suggest that salidroside attenuates hypoxia-induced renal injury through modulation of intrarenal dopamine signaling and highlight its potential as a preventive or therapeutic agent for individuals exposed to hypobaric hypoxia.

## 1. Introduction

Individuals who have lived in lowland areas for extended periods are prone to acute high-altitude illness upon rapid ascent to elevations above 3,000 meters, with an incidence as high as 45%−85% [1–3]. The kidney, a vital organ for maintaining internal homeostasis, is highly sensitive to hypoxia. In addition to the lungs and brain, it is among the organs most vulnerable to hypobaric hypoxia [4,5]. As a result, the unique environmental conditions at high altitude may cause proteinuria or even AKI in some individuals who ascend rapidly, thereby posing a serious threat to public health and safety.

Rhodiola exhibits multiple pharmacological effects, including scavenging reactive oxygen species (ROS) [6], reducing intracellular calcium overload [6,7], and inhibiting apoptosis [8,9]. Additionally, it can alleviate hypoxic injury by upregulating hypoxia-inducible factor-1α (HIF-1α) and its downstream target, vascular endothelial growth factor (VEGF) [10]. These properties make it widely used for the prevention and treatment of acute high-altitude illness. Salidroside (chemical name: 2-(4-hydroxyphenyl)ethyl-β-D-glucopyranoside; molecular formula: C₁₄H₂₀O₇) is a major bioactive compound extracted from Rhodiola. Previous studies have demonstrated that salidroside exerts protective effects against AKI of various etiologies [11–14]. However, its potential role in preventing or mitigating high-altitude-induced kidney injury has not yet been confirmed.

Intrarenal dopamine is an important regulatory factor for maintaining normal renal function [15]. It modulates renal hemodynamics [16], inhibits the reabsorption of salt and water, counteracts the renin-angiotensin system, and suppresses oxidative stress [16,17]. Its renoprotective effects have been demonstrated in various models of kidney injury [15]. Dopamine exerts its biological functions through dopamine receptors, whose phosphorylation is primarily regulated by G protein-coupled receptor kinases (GRKs). Among the GRK subtypes, GRK4 has been shown to be predominantly expressed in renal tissues [18]. Upregulation of GRK4 expression and activity leads to excessive phosphorylation of renal DRD1, contributing to elevated blood pressure [18,19] and aggravation of acute renal ischemia/reperfusion (I/R) injury. In contrast, downregulation of GRK4 has been found to protect the kidney from I/R-induced damage [20]. In a model of high-altitude hypoxia-induced brain injury, salidroside was reported to improve dopaminergic dysfunction and reduce oxidative stress, thereby exerting neuroprotective effects [21–24]. However, whether salidroside confers renal protection by modulating the intrarenal dopamine signaling pathway under hypoxic conditions remains to be elucidated.

**Fig 1:**
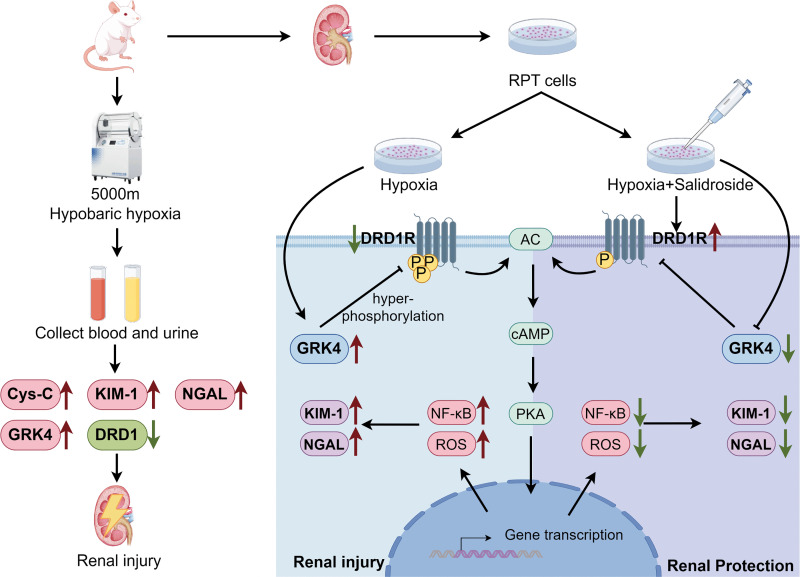
Schematic model of salidroside-mediated protection against hypoxia-induced kidney injury. Proposed mechanism illustrating how salidroside attenuates hypoxia-induced renal injury through regulation of DRD1 and GRK4 signaling.

Therefore, this study aimed to establish a simulated high-altitude environment by housing rats in a hypobaric chamber. In parallel, RPT cells were cultured under hypoxic conditions. The extent of kidney injury in rats exposed to the simulated high-altitude environment was assessed. Furthermore, based on the intrarenal dopamine system, this study explored the role and underlying mechanisms of salidroside in the prevention and treatment of high-altitude-induced kidney injury. The proposed mechanism is schematically illustrated in Fig 1.

## 2. Material and methods

### 2.1. In vivo experiments

#### 2.1.1. Animals and grouping.

SPF-grade male Sprague-Dawley (SD) rats (7–8 weeks old, weighing 170 ± 20 g) were obtained from Ensiweier Biotechnology Co., Ltd. (Chengdu, China; license number: SCXK (Xiang) 2019–0004). After one week of acclimatization, rats were randomly assigned to five groups: control (n = 30), hypoxia model (n = 30), and low-, medium-, and high-dose salidroside intervention groups (n = 30 per group). A hypobaric chamber (YuYan, LP-1500, Shanghai, China) was used to simulate a high-altitude environment of 5000 m. Rats in the model and salidroside groups were housed in the chamber under controlled conditions (22–25 °C, 50% relative humidity, 47–52 kPa), with free access to food and water and a 12-hour light/dark cycle. Control rats were housed under normobaric conditions (500 m altitude, 70–80% humidity). Rats in the intervention groups received daily intragastric salidroside (20, 40, or 60 mg/kg/day; Yuanye, S25475-5g, Shanghai, China). Control and model groups received equal volumes of 0.9% saline (Kelun Pharmaceutical, Sichuan, China). Except for daily dosing and feeding, rats in the model and intervention groups remained in the hypobaric chamber throughout the experiment.

#### 2.1.2. Collection of urine, blood, and kidney tissue samples.

Urine samples were collected from rats housed individually in metabolic cages (Junwei Instruments, Shanghai, China) on days 0, 1, 3, 7, 14, and 28 of the experimental period. Following urine collection, all rats were anesthetized with 1% sodium pentobarbital solution (50 mg/kg; Sigma, P3761, USA) administered intraperitoneally. After complete loss of reflexes was confirmed, blood samples were obtained from the abdominal aorta for the measurement of kidney injury-related biomarkers. The animals were euthanized by decapitation approximately 30 min after anesthesia induction. A portion of the left kidney was placed in cryogenic tubes, rapidly frozen in liquid nitrogen, and stored at −80 °C for Western blot analysis. The right kidney was fixed in 4% paraformaldehyde (Biosharp, BL539A, China) for histological and immunohistochemical analyses.

#### 2.1.3. Optical microscopic examination of renal tissue.

After embedding and sectioning the kidney tissues, hematoxylin-eosin (HE) staining (Solarbio, G1120, China) and periodic acid-Schiff (PAS) staining (Solarbio, G1281, China) were conducted. Subsequently, the stained sections were imaged using a standard light microscope (Leica, Germany).

#### 2.1.4. Measurement of serum NGAL, KIM-1, and Cys-C, and urinary NGAL and KIM-1 levels by ELISA.

The concentrations of NGAL (JM-01678R1), KIM-1 (JM-01724R1), and Cys-C (JM-01845R1) in rat serum and urine were determined using enzyme-linked immunosorbent assay (ELISA) kits (Jiangsu Jingmei, China).

#### 2.1.5. Immunohistochemistry.

Kidney tissues were paraffin-embedded and sectioned at a thickness of 5 μm. The sections were subsequently dewaxed and subjected to antigen retrieval. After blocking with 5% goat serum (Solarbio, SL038), the samples were incubated overnight at 4 °C with primary antibodies, including rabbit anti-DRD1 (Abcam, ab279713, UK, 1:200) and mouse anti-GRK4 (Santa Cruz, sc-9985, USA, 1:200). The next day, the sections were incubated for 20 minutes at 37 °C with secondary antibodies: goat anti-mouse IgG H&L (AF488, 550036, China, 1:2000) and goat anti-rabbit IgG H&L (AF594, 550043, China, 1:2000). DAB staining and hematoxylin counterstaining were then performed, followed by mounting of the slides. Finally, the stained sections were examined using a conventional optical microscope (Leica, Germany) and quantitatively analyzed with ImageJ software (USA).

#### 2.1.6. Immunoblotting.

Kidney tissues were lysed using RIPA buffer (Solarbio, R0010, China), and total protein concentrations were measured with the BCA assay (Solarbio, PC0020, China). Equal amounts of protein were separated by sodium dodecyl sulfate-polyacrylamide gel electrophoresis (SDS-PAGE) and subsequently transferred onto PVDF membranes (Roche, 18071300, China). The membranes were then blocked with 3% BSA (BIOFROXX, 4240GR250, Germany) for 1 hour and incubated overnight at 4 °C with primary antibodies: rabbit anti-DRD1 (Abcam, ab279713, UK, 1:400) and mouse anti-GRK4 (Santa Cruz, sc-9985, USA, 1:200). On the following day, membranes were incubated for 1 hour at room temperature with HRP-conjugated secondary antibodies (Goat Anti-rabbit IgG H&L, 511203, China, 1:10000; Goat Anti-mouse IgG H&L, 511103, China, 1:10000). Protein bands were visualized using chemiluminescence, and band intensities were quantified using ImageJ software. GAPDH was used as a loading control for normalization. Uncropped Western blot images corresponding to Fig 5, Fig 6, and Fig 14 are provided in S1–S3 Figs.

### 2.2. Molecular docking

The three-dimensional structures of the target proteins GRK4 and DRD1 were retrieved from the RCSB Protein Data Bank (https://www.rcsb.org/). Redundant ligands were removed using PyMOL 2.5, and the cleaned structures were imported into AutoDock Vina. Water molecules were removed, hydrogen atoms were added, and the resulting structures were saved as PDBQT files for use as receptor models. The molecular structure of salidroside was downloaded from the PubChem database (https://pubchem.ncbi.nlm.nih.gov/) and converted into PDB format. Molecular docking was then carried out using AutoDock Vina, and the docking results were visualized using PyMOL 2.5.

### 2.3. In vitro experiments

RPT cells were isolated from the kidney tissue of Sprague-Dawley (SD) rats and cultured in DMEM/F12 complete medium. The medium was supplemented with recombinant human epidermal growth factor (10 ng/ml), 1 × insulin-transferrin-selenium, dexamethasone (4 mg/ml), penicillin-streptomycin (1%), and fetal bovine serum (10%). Cells were incubated at 37 °C in a 5% CO₂ atmosphere. According to the experimental design, cells were divided into six groups: Con (normoxia), Sal 1 (normoxia + 25 μM salidroside), Hypoxia, Sal 2 (hypoxia + 25 μM salidroside), Fen + Sal (hypoxia + 1 μM fenoldopam + 25 μM salidroside), and siDRD1 + Sal (hypoxia + siDRD1 + 25 μM salidroside). Normoxic cultures were maintained in an incubator with 5% CO₂ at 37 °C, whereas hypoxic cultures were maintained in an incubator with 5% CO₂ and 1% O₂. After 24 hours of incubation, subsequent assays were performed.

#### 2.3.1. Determination of RPT cell viability.

Primary RPT cells were seeded into 96-well plates. When the cells reached 80−90% confluence, the culture medium was replaced with complete medium containing salidroside at final concentrations of 0, 3.125 µM, 6.25 µM, 12.5 μM, 25 µM, 50 µM, 100 µM, or 200 µM. The cells were then incubated for 24 hours under either normoxic or hypoxic conditions. Cell viability was subsequently assessed using the CCK-8 assay.

#### 2.3.2. Measurement of NGAL and KIM-1 levels in cell culture supernatants by ELISA.

RPT cells were cultured according to the procedure described in Method 2.3.1. After treatment, cell culture supernatants from each group were collected, and the concentrations of NGAL (JM-01678R2, Jiangsu Jingmei, China) and KIM-1 (JM-01724R2, Jiangsu Jingmei, China) were quantified using enzyme-linked immunosorbent assay (ELISA).

#### 2.3.3. Quantification of GRK4 and DRD1 mRNA expression in RPT cells.

Total RNA was extracted from RPT cells using Trizol reagent (Takara, 9109, Japan), and reverse transcription was performed with the RT OR-Easy^TM^ II kit (FOREGENE, 220303, China) to synthesize cDNA. Quantitative analysis of GRK4 and DRD1 mRNA expression was conducted using a fully automated medical PCR analysis system (SLAN-96S, Shanghai Hongshi Medical Technology Co., Ltd., China). GAPDH was used as an internal control for normalization. Primers used in this study were designed and synthesized by Sangon Biotech Co., Ltd. (China), and their sequences were as follows:

GRK4-F GTCACCATTCCCTGGCAGAA
GRK4-R CTCCTCACTGTGGGCAATGT
DRD1-F CACCTGAGGTCCAAGGTGAC
DRD1-R AAGGACCCAAAGGGCCAAAA
R-GAPDH-F GGTGAAGGTCGGTGTGAACG
R-GAPDH-R CTCGCTCCTGGAAGATGGTG

The experimental results were analyzed using the comparative cycle threshold (ΔΔCT) method to determine relative gene expression levels.

#### 2.3.4. DRD1-siRNA transfection in RPT cells.

RPT cells were transferred to antibiotic-free DMEM/F12 medium 24 hours prior to transfection. DRD1-siRNA (100 pmol) and Lip2000 (5 μL; Biosharp, BL623A, Beijing) were each diluted in 250 μL of serum-free medium, then combined to form transfection complexes, which were subsequently added to the cells. After incubation at 37 °C for 4–6 hours, the medium was replaced with complete culture medium, and the cells were maintained for an additional 48 hours before being used in subsequent experiments.

#### 2.3.5. Quantification of GRK4 and DRD1 protein expression in RPT cells.

Cellular immunoblotting was performed following the protocol described in section 2.1.6. Primary antibodies included rabbit anti-DRD1 (Abcam, ab279713, 1:200) and mouse anti-GRK4 (Santa Cruz, sc-9985, 1:200). The corresponding secondary antibodies were Goat anti-rabbit IgG H&L (511203, 1:1000) and Goat anti-mouse IgG H&L (511103, 1:10000).

#### 2.3.6. Detection of apoptosis in each group of cells by flow cytometry.

RPT cells were collected and washed twice with PBS, then resuspended in 100 μL of 1 × Binding Buffer. Annexin V-FITC (5 μL) and PI (10 μL; Yeasen, 40303ES60, Shanghai) were added sequentially, and the mixture was incubated in the dark for 10−15 minutes. After incubation, 40 μL of 1 × Binding Buffer was added to each sample. The stained cells were immediately analyzed using a flow cytometer (CytoFLEX, Beckman Coulter, China), and apoptosis rates were quantified using FlowJo^TM^ v10 software (USA).

### 2.4. Ethics statement

All animal procedures were performed in strict accordance with the Guidelines for the Care and Use of Laboratory Animals issued by the Chinese Medical Research Council and were approved by the Animal Welfare Committee of the PLA Western Theater General Hospital (approval number: 2023EC3-ky011). The study complied with the ARRIVE guidelines and followed the recommendations in the AVMA Guidelines for the Euthanasia of Animals (2020). Every effort was made to minimize the number of animals used and to reduce pain or distress during the experiments.

### 2.5. Statistical analysis

Statistical analyses were performed using SPSS 25.0 and GraphPad Prism 9.0. Experimental data were presented as means ± standard deviation (Mean ± SD) for continuous variables that followed a normal distribution. For comparisons among multiple groups, one-way analysis of variance (one-way ANOVA) was performed when both normality and homogeneity of variance assumptions were met, with Bonferroni post hoc tests used for pairwise comparisons. If the data did not meet the assumptions of normality or homogeneity of variance, the Kruskal-Wallis H test was applied to compare medians among groups, followed by Mann-Whitney U tests for pairwise comparisons. In this case, *P* values were adjusted using the Bonferroni correction method. Statistical significance was defined as an adjusted *P* value < 0.05.

## 3. Experimental results

### 3.1. In vivo experimental results

#### 3.1.1. Histopathological changes of renal tissues in each group of rats.

**Fig 2:**
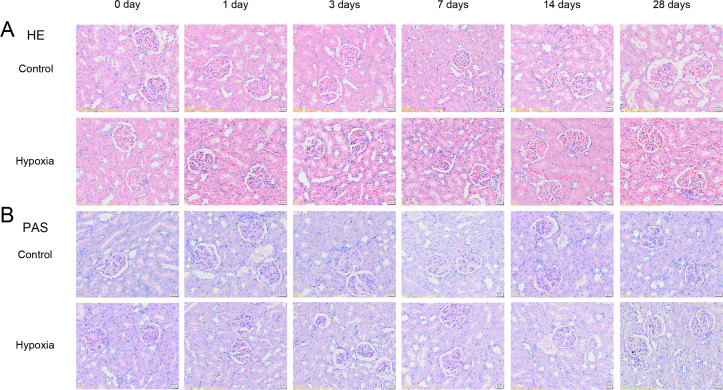
Histopathological examination of renal tissues. **(A)** Hematoxylin-eosin (HE) staining of kidney tissue (×400). **(B)** Periodic acid-Schiff (PAS) staining of kidney tissue (×400).

Hematoxylin and eosin (HE) staining of kidney tissues revealed tubulointerstitial congestion in experimental rats on days 14 and 28 (Fig 2A). Periodic acid-Schiff (PAS) staining showed focal swelling of renal tubular epithelial cells in the same groups on days 14 and 28 (Fig 2B).

#### 3.1.2. KIM-1 levels in blood and urine samples of rats from each group.

**Fig 3:**
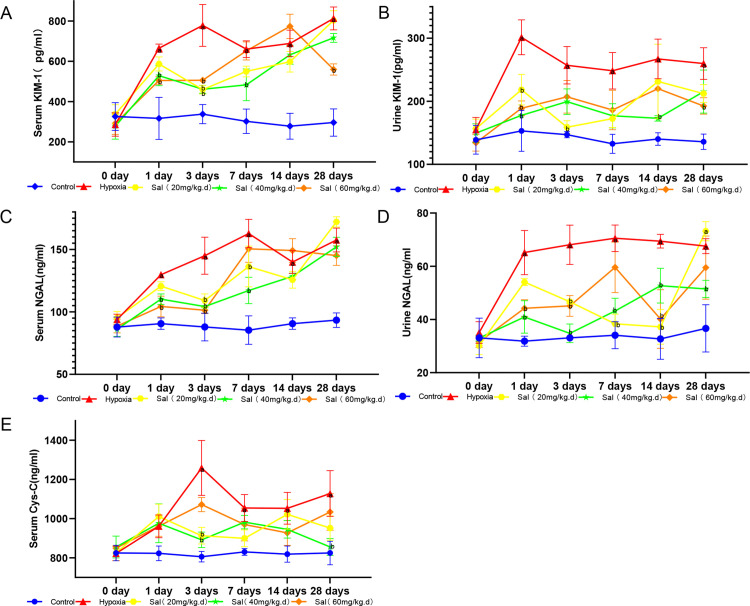
Salidroside attenuates renal injury induced by hypobaric hypoxia. **(A)** Serum KIM-1 levels measured by ELISA. **(B)** Urinary KIM-1 levels measured by ELISA. **(C)** Serum NGAL levels measured by ELISA. **(D)** Urinary NGAL levels measured by ELISA. **(E)** Serum Cys-C levels measured by ELISA. Data are presented as mean ± SD (n = 5 per group).a *P* < 0.05 vs. control group at the same time point; b *P* < 0.05 vs. hypoxia group. Control: control group; Hypoxia: hypoxia group; Sal (20 mg/kg·d): low-dose salidroside group; Sal (40 mg/kg·d): medium-dose salidroside group; Sal (60 mg/kg·d): high-dose salidroside group.

Serum KIM-1 levels were significantly higher in the experimental group than in the control group on days 1, 3, 7, 14, and 28 (*P* < 0.0001). On day 3, the low-dose salidroside group exhibited significantly lower serum KIM-1 levels compared to the experimental group (*P* = 0.0011). Serum KIM-1 levels were also significantly lower in the medium-dose salidroside group compared to the experimental group on days 1 (*P* = 0.0281), 3 (*P* = 0.0006), and 7 (*P* = 0.0129). Similarly, the high-dose salidroside group showed significantly lower serum KIM-1 levels than the experimental group on days 1 (*P* = 0.0004), 3 (*P* = 0.0046), and 28 (*P* = 0.0007) (Fig 3A).

Urinary KIM-1 levels were significantly higher in the experimental group than in the control group on days 1 (*P* < 0.0001), 3 (*P* = 0.0006), 7 (*P* = 0.0035), 14 (*P* = 0.0001), and 28 (*P* = 0.0012). In the low-dose salidroside intervention group, urinary KIM-1 were significantly lower than in the experimental group on days 1 (*P* = 0.0056) and 3 (*P* = 0.0026). Similarly, in the medium-dose group, levels were significantly reduced on days 1 (*P* < 0.0001) and 14 (*P* = 0.0109), compared to the experimental group. In the high-dose group, urinary KIM-1 levels were significantly reduced on day 1 (*P* < 0.0001) relative to the experimental group (Fig 3B).

#### 3.1.3. NGAL levels in blood and urine samples of rats from each group.

Serum NGAL levels in the experimental group were significantly higher than those in the control group on days 1, 3, 7, 14, and 28 (*P* < 0.0001). Treatment with low-dose salidroside significantly reduced serum NGAL levels compared to the experimental group on days 3 (*P* = 0.0424) and 7 (*P* = 0.0007). In the medium-dose group, significant reductions were observed on days 1 (*P* = 0.0137), 3 (*P* < 0.0001), and 7 (*P* < 0.0001). Similarly, the high-dose group exhibited markedly lower serum NGAL levels than the experimental group on days 1 (*P* = 0.0037) and 3 (*P* < 0.0001) (Fig 3C).

Urinary NGAL levels were significantly higher in the experimental group than in the control group on days 1, 3, 7, 14, and 28 (*P* < 0.0001). Administration of low-dose salidroside significantly reduced urinary NGAL levels compared to the experimental group on days 3 (*P* = 0.0035), 7 (*P* < 0.0001), and 14 (*P* = 0.0003). The medium-dose group showed significant reductions on all five time points—days 1 (*P* = 0.0014), 3 (*P* < 0.0001), 7 (*P* < 0.0001), 14 (*P* = 0.0043), and 28 (*P* = 0.0020)—relative to the experimental group. Similarly, the high-dose group exhibited significantly lower urinary NGAL levels on days 1 (*P* = 0.0396), 3 (*P* = 0.0003), and 14 (*P* < 0.0001) when compared with the experimental group (Fig 3D).

#### 3.1.4. Serum Cys-C levels in each group of rats.

Serum Cys-C levels were significantly higher in the experimental group than in the control group on days 3 (*P* < 0.0001), 7 (*P* = 0.0064), 14 (*P* = 0.0045), and 28 (*P* = 0.001). On day 3, the low-dose salidroside group exhibited significantly lower serum Cys-C levels compared to the experimental group (*P* = 0.0013). In the medium-dose group, significant reductions were also observed on days 3 (*P* = 0.0002) and 28 (*P* = 0.003). However, the high-dose group did not show any significant difference in serum Cys-C levels relative to the experimental group (*P* > 0.05) (Fig 3E). The raw data underlying Fig 3 are provided in S1 Table.

#### 3.1.5. The changes in expression of GRK4 and DRD1 in renal tissues of each rat group by immunohistochemistry.

**Fig 4:**
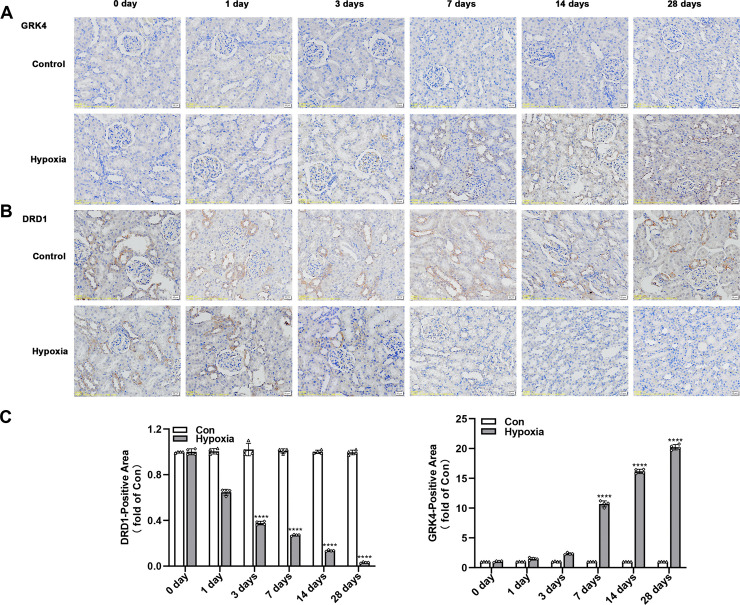
Immunohistochemical analysis of GRK4 and DRD1 protein expression in rat kidneys. **(A)** GRK4 protein expression in renal tissue (×400, n = 5); **(B)** DRD1 protein expression in renal tissue (×400, n = 5); **(C)** Semi-quantitative analysis of GRK4 and DRD1 expression in the hypoxia and medium-dose salidroside groups. All data are expressed as mean ± SD. **** *P* < 0.0001 vs. control group. Control: control group; Hypoxia: hypoxia group.

GRK4 and DRD1 proteins were predominantly localized in the renal tubules, with only minimal expression observed in the glomeruli (Fig 4A and 4B). With increasing duration of hypobaric hypoxia exposure, GRK4 expression in the kidneys of experimental rats gradually increased, showing a marked elevation by day 7 and peaking at day 28. In contrast, DRD1 expression began to decline significantly from day 3 and progressively decreased, reaching its lowest level at day 28 (Fig 4C).

#### 3.1.6. The expression levels of DRD1 and GRK4 proteins in renal tissues by Western blot.

**Fig 5:**
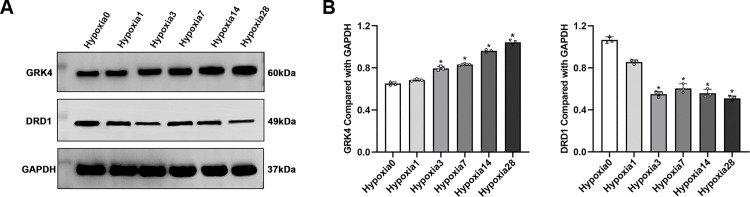
Western blot analysis of GRK4 and DRD1 protein expression after hypoxia exposure. The relative expression levels of GRK4 and DRD1 proteins in rat kidneys were determined by Western blot. Data are presented as mean ± SD from at least three independent experiments.* *P* < 0.05, ** *P* < 0.01, **** *P* < 0.0001 vs. day 0 group. Hypoxia: hypoxia group on day **X.** (n = 3).

**Fig 6:**
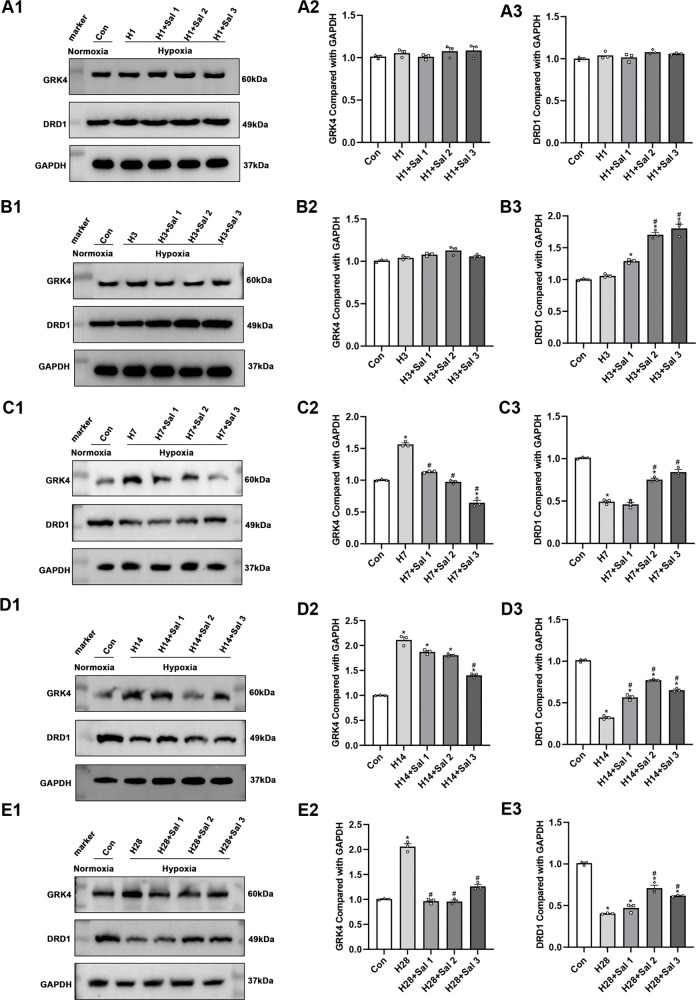
Western blot analysis of GRK4 and DRD1 protein expression following salidroside treatment. (A1-A3) Expression after 1 day of hypobaric hypoxia. (B1-B3) 3 days. (C1-C3) 7 days. (D1-D3) 14 days. (E1-E3) 28 days. Data are presented as mean ± SD from at least three independent experiments (n = 3). * *P* < 0.05 vs. control group; # *P* < 0.05 vs. hypoxia group. HX: hypoxia group on day X; HX + Sal1: low-dose salidroside group on day X; HX + Sal2: medium-dose salidroside group on day X; HX + Sal3: high-dose salidroside group on day **X.**

Compared to the control group, GRK4 protein expression in the renal tissues of rats in the experimental group was significantly increased on days 3, 7, 14, and 28 following hypoxic exposure (*P* < 0.05), exhibiting a time-dependent upward trend. In contrast, DRD1 protein expression in the same tissues was significantly reduced on days 7, 14, and 28 (*P* < 0.05), demonstrating a progressive decline with prolonged hypoxia (Fig 5). Following salidroside intervention, DRD1 expression was elevated in the medium- and high-dose groups compared to the experimental group on day 3 (Fig 6 B1-B3). On day 7, GRK4 expression was reduced across all salidroside-treated groups, while DRD1 expression remained elevated in the medium- and high-dose groups (Fig 6 C1-C3). By day 14, GRK4 expression was significantly lower in the high-dose group, and DRD1 expression was increased in all salidroside-treated groups (Fig 6 D1-D3). On day 28, GRK4 expression continued to be suppressed in the low-, medium-, and high-dose groups, whereas DRD1 expression remained elevated in the medium- and high-dose groups (Fig 6 E1-E3).

### 3.2. Molecular docking results

**Fig 7:**
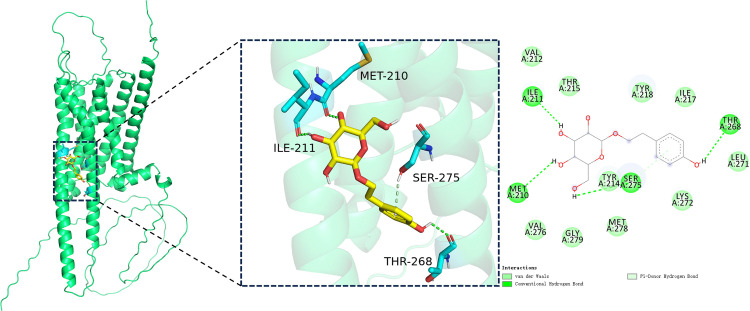
Molecular docking analysis of salidroside with DRD1. Left to right: overall 3D docking model, local 3D interaction view, and 2D interaction diagram. Green represents the DRD1 protein, cyan indicates interacting amino acid residues, and yellow denotes salidroside.

**Fig 8:**
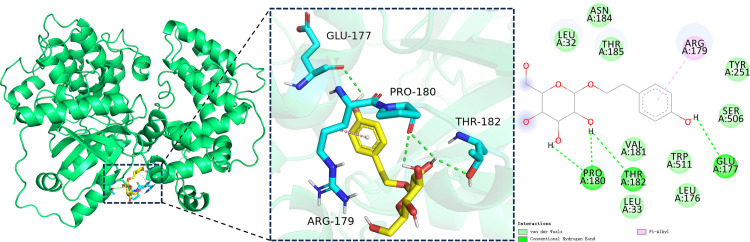
Molecular docking analysis of salidroside with GRK4. Left to right: overall 3D docking model, local 3D interaction view, and 2D interaction diagram. Green represents the GRK4 protein, cyan indicates interacting amino acid residues, and yellow denotes salidroside.

The minimum binding energies of salidroside binding to DRD1 and GRK4 were –6.3 kcal/mol and–6.9 kcal/mol, respectively. Both values are below –5 kcal/mol, indicating strong and favorable binding affinities (Fig 7 and Fig 8).

### 3.3. Results of in vitro experiments

#### 3.3.1. Effect of salidroside on the viability of RPT cells.

**Fig 9:**
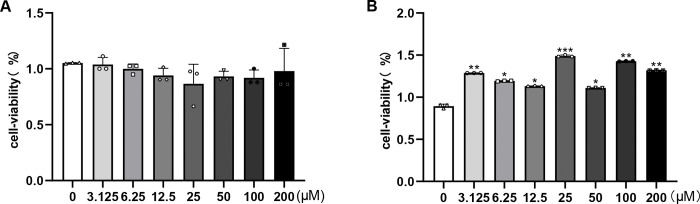
Effects of salidroside on RPT cell viability under normoxic and hypoxic conditions. **(A)** Cell viability of RPT cells under normoxia following salidroside treatment. **(B)** Cell viability of RPT cells under hypoxia following salidroside treatment, assessed by CCK-8 assay. * *P* < 0.05, ** *P* < 0.01, *** *P* < 0.0001 vs. control group. Normoxia: normoxic condition; Hypoxia: hypoxic condition.

Under normoxic conditions, pretreatment with salidroside at concentrations of 0, 3.125 μM, 6.25 μM, 12.5 μM, 25 μM, 50 μM, 100 μM, or 200 μM for 24 hours did not significantly affect RPT cell viability (Fig 9A). In contrast, under hypoxic conditions, 24-hour salidroside pretreatment markedly enhanced cell viability, with the greatest effect observed at 25 μM. Increasing the concentration beyond this point did not lead to further improvements. Therefore, 25 μM salidroside was chosen for subsequent experiments (Fig 9B).

#### 3.3.2. The expression levels of DRD1 mRNA in RPT cells transfected with siDRD1.

**Fig 10:**
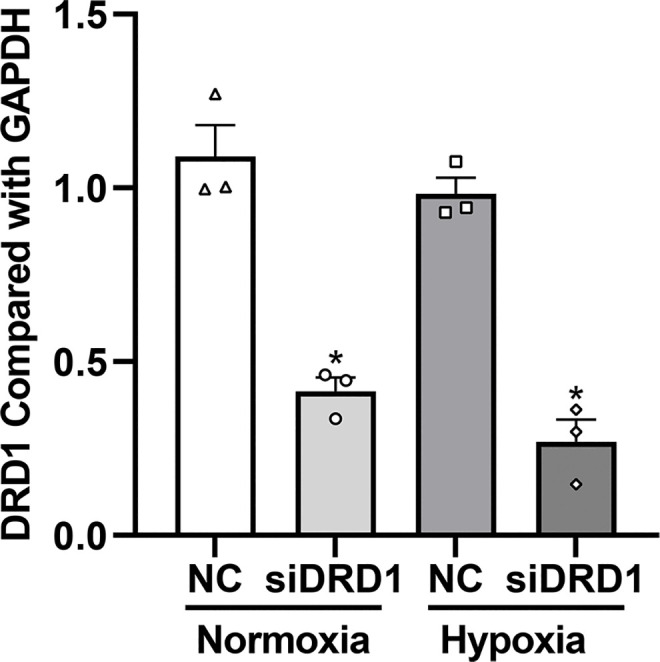
qRT-PCR analysis of DRD1 gene expression in RPT cells transfected with siDRD1. DRD1 mRNA expression was significantly reduced in siDRD1-transfected cells compared with the non-specific control group. Data are presented as mean ± SD (n = 3).* *P* < 0.05 vs. non-specific control group. NC: non-specific control group; siDRD1: transfection group; Normoxia: normoxic condition; Hypoxia: hypoxic condition.

DRD1 mRNA expression was significantly reduced in RPT cells transfected with siDRD1 compared to the non-transfected group under both normoxic and hypoxic conditions (*P* < 0.05), confirming that siDRD1 effectively suppressed DRD1 mRNA expression (Fig 10).

#### 3.3.3. The effects of salidroside on NGAL and KIM-1 expression in each group of RPT cells.

**Fig 11:**
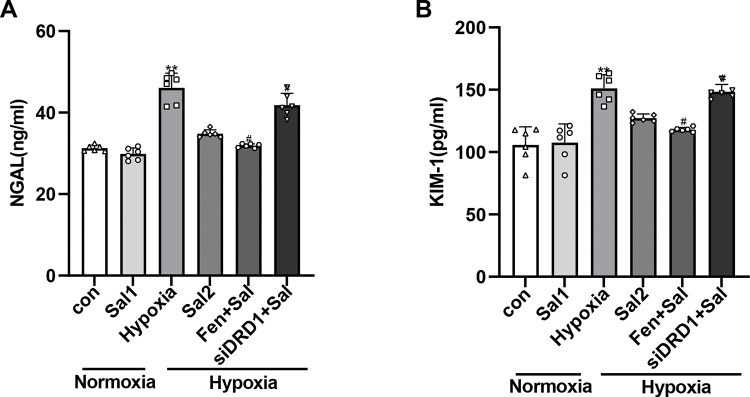
Effects of salidroside on RPT cell injury under hypoxic conditions. (A) NGAL secretion in RPT cell supernatants. (B) KIM-1 secretion in RPT cell supernatants. * *P* < 0.01 vs. normoxia group; # *P* < 0.05 vs. hypoxia + salidroside group. Con: normoxia group; Sal 1: normoxia + 25 μM salidroside; Hypoxia: hypoxia group; Sal 2: hypoxia + 25 μM salidroside; Fen + Sal: hypoxia + 1 μM fenoldopam + 25 μM salidroside; siDRD1 + Sal: hypoxia + siDRD1 + 25 μM salidroside.

The levels of NGAL and KIM-1 were significantly elevated in the hypoxia group compared to the normoxia group (*P* < 0.05), indicating renal tubular injury induced by hypoxic stress. Treatment with salidroside for 24 hours under hypoxic conditions markedly suppressed the secretion of both biomarkers (*P* < 0.05), suggesting a protective effect. Notably, co-treatment with fenoldopam and salidroside under hypoxia further reduced NGAL and KIM-1 levels in the culture supernatant compared to salidroside treatment alone (*P* < 0.05), implying that DRD1 activation may enhance the protective effects of salidroside. Conversely, in hypoxia-exposed RPT cells with DRD1 knockdown, the protective effect of salidroside was diminished, as reflected by increased NGAL and KIM-1 levels relative to the salidroside-only group (*P* < 0.05). Collectively, these findings indicate that the renoprotective action of salidroside under hypoxic conditions is at least partially mediated through DRD1 signaling (Fig 11). The raw data underlying Fig 11 are provided in S2 Table.

#### 3.3.4. Effect of Salidroside on apoptosis in each group of RPT Cells.

**Fig 12:**
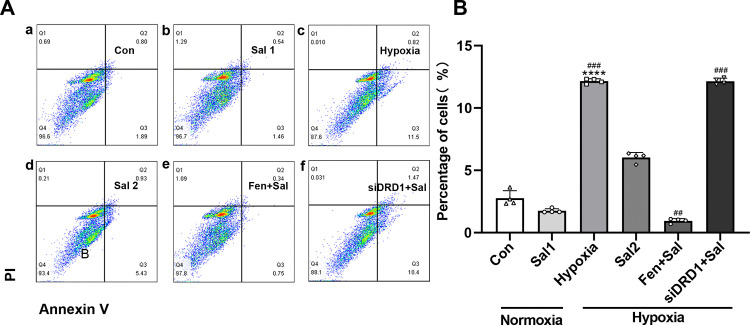
Flow cytometry analysis of the effects of salidroside on apoptosis in RPT cells. (A) Flow cytometric detection of apoptosis in RPT cells. (B) Quantitative analysis of relative apoptosis levels. **** *P* < 0.0001 vs. normoxia group; ## *P* < 0.01, ### *P* < 0.001 vs. hypoxia + salidroside group. Con: normoxia group; Sal 1: normoxia + 25 μM salidroside; Hypoxia: hypoxia group; Sal 2: hypoxia + 25 μM salidroside; Fen + Sal: hypoxia + 1 μM fenoldopam + 25 μM salidroside; siDRD1 + Sal: hypoxia + siDRD1 + 25 μM salidroside.

A 24-hour hypoxic culture significantly increased the percentage of apoptotic RPT cells compared to the control group (*P* < 0.0001), with early apoptosis being the predominant form. Salidroside treatment markedly reduced the apoptosis rate relative to the hypoxia group (*P* < 0.001). Furthermore, co-treatment with fenoldopam and salidroside resulted in a further decrease in apoptosis compared to salidroside treatment alone (*P* < 0.01). Conversely, DRD1 knockdown followed by salidroside administration significantly increased apoptosis compared to salidroside alone (*P* < 0.001) (Fig 12).

#### 3.3.5. The expression levels of DRD1 and GRK4 mRNA in each group of RPT cells.

**Fig 13:**
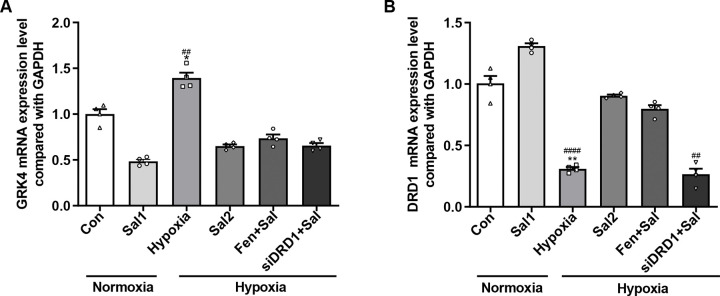
Quantitative real-time PCR analysis of GRK4 and DRD1 mRNA expression in RPT cells. **(A)** GRK4 mRNA expression. **(B)** DRD1 mRNA expression. * *P* < 0.05, ** *P* < 0.01 vs. normoxia group; ## *P* < 0.01, #### *P* < 0.0001 vs. hypoxia + salidroside group. Con: normoxia group; Sal 1: normoxia + 25 μM salidroside; Hypoxia: hypoxia group; Sal 2: hypoxia + 25 μM salidroside; Fen + Sal: hypoxia + 1 μM fenoldopam + 25 μM salidroside; siDRD1 + Sal: hypoxia + siDRD1 + 25 μM salidroside.

The expression levels of GRK4 and DRD1 mRNA in RPT cells were evaluated using qRT-PCR. Compared to the normoxia group, hypoxic conditions significantly increased GRK4 mRNA expression (*P* < 0.05) and decreased DRD1 mRNA expression (*P* < 0.01). Treatment with salidroside under hypoxia led to a significant reduction in GRK4 mRNA (*P* < 0.01) and a marked elevation in DRD1 mRNA levels (*P* < 0.0001) compared to the hypoxia group. Notably, co-treatment with fenoldopam and salidroside did not produce significant differences in GRK4 or DRD1 mRNA levels compared to the salidroside-only group. In contrast, DRD1 knockdown followed by salidroside treatment significantly reduced DRD1 mRNA expression relative to the salidroside-only group (*P* < 0.01), while GRK4 mRNA levels remained unchanged (*P* > 0.05) (Fig 13).

#### 3.3.6. DRD1 and GRK4 protein expression levels in each group of RPT cells.

**Fig 14:**
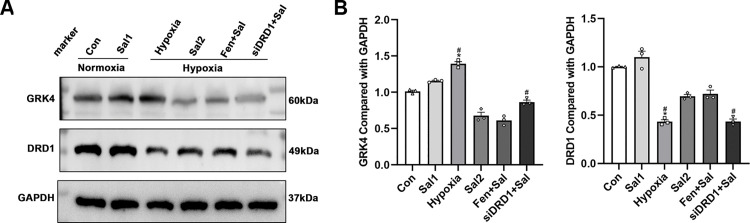
Western blot analysis of GRK4 and DRD1 protein expression in RPT cells. (A) Representative Western blot bands for GRK4 and DRD1 across experimental groups. (B) Quantitative analysis of relative protein expression. ** *P* < 0.01, *** *P* < 0.001 vs. normoxia group; ## *P* < 0.01, ### *P* < 0.001 vs. hypoxia + salidroside group. Con: normoxia group; Sal 1: normoxia + 25 μM salidroside; Hypoxia: hypoxia group; Sal 2: hypoxia + 25 μM salidroside; Fen + Sal: hypoxia + 1 μM fenoldopam + 25 μM salidroside; siDRD1 + Sal: hypoxia + siDRD1 + 25 μM salidroside.

GRK4 and DRD1 protein expression levels in RPT cells were analyzed by Western blot. Compared to the normoxia group, hypoxia-treated RPT cells showed a significant increase in GRK4 protein expression (*P* < 0.01) and a significant decrease in DRD1 protein expression (*P* < 0.001). In cells treated with salidroside under hypoxic conditions for 24 hours, GRK4 protein expression was markedly reduced (*P* < 0.001), while DRD1 protein expression was significantly elevated (*P* < 0.01) compared to the hypoxia-only group. Notably, co-treatment with fenoldopam and salidroside under hypoxia did not produce significant changes in GRK4 or DRD1 protein levels compared to salidroside alone (*P* > 0.05). In contrast, DRD1 knockdown followed by salidroside treatment under hypoxia led to a significant increase in GRK4 expression (*P* < 0.05) and a notable decrease in DRD1 expression (*P* < 0.01), relative to the hypoxia plus salidroside group (Fig 14).

## 4. Discussion

This study employed a hypobaric hypoxia chamber to simulate a 5,000-meter plateau environment and explored kidney injury in SD rats under acute hypobaric hypoxia, along with the potential protective effects of salidroside.

NGAL, KIM-1, and Cys-C are commonly used biomarkers for predicting the severity of kidney injury and monitoring the progression of renal diseases [25–29]. In the hypoxia-exposed group, serum and urinary levels of NGAL and KIM-1 were significantly elevated after just one day, whereas serum Cys-C levels began to rise on day 3. Histopathological changes, such as tubulointerstitial edema and vascular congestion, became evident after 14 and 28 days of exposure. Collectively, these findings suggest that hypobaric hypoxia induces renal injury in rats, with NGAL and KIM-1 acting as early and sensitive indicators of kidney damage [29].

Previous studies have demonstrated that Rhodiola exerts protective effects against kidney injuries caused by various factors, including exhaustive exercise, cisplatin administration, sepsis, and severe pancreatitis. These renoprotective effects—particularly those attributed to salidroside, the main active component—are thought to be mediated through its antioxidant, anti-inflammatory, and anti-apoptotic properties [11–14,30,31]. In the present study, rats exposed to hypobaric hypoxia were administered salidroside intragastrically at varying doses. The findings revealed that salidroside at 20−60 mg/kg·d effectively attenuated hypoxia-induced renal injury, as evidenced by decreased levels of Cys-C, KIM-1, and NGAL.

The underlying mechanisms of kidney injury induced by hypobaric hypoxia at high altitude remain incompletely understood. Renal dysfunction in such environments is thought to result from both the direct effects of hypoxia on renal tissues and a range of compensatory physiological responses, including alterations in ventilation, cardiac output, sympathetic nervous system activity, and erythropoiesis [32,33]. Emerging evidence suggests that the renal local dopamine system contributes to the pathogenesis of kidney injury. In particular, activation of the DRD1 has been shown to exert renoprotective effects. For example, selective DRD1 agonists protect podocytes in diabetic mice from apoptosis and oxidative stress [34]. The function of renal DRD1 is tightly regulated by its phosphorylation status, which is mediated by GRK4. Elevated GRK4 expression or activity leads to DRD1 hyperphosphorylation, disrupting G protein coupling and impairing DRD1 function. Recent studies have demonstrated that GRK4 overexpression inhibits DRD1 activity and exacerbates acute renal I/R injury, whereas GRK4 knockout enhances DRD1 signaling and mitigates such damage [35]. In our study, under simulated high-altitude conditions, we observed increased GRK4 expression and decreased DRD1 expression in renal tissues, accompanied by elevated levels of Cys-C, KIM-1, and NGAL. These molecular changes are likely associated with the development of AKI under hypobaric hypoxia. To explore whether salidroside, a traditional agent known to enhance adaptation to high-altitude environments, exerts renoprotective effects by modulating the renal local dopamine system, we first performed molecular docking studies. The results showed that salidroside had a minimum docking score of –6.3 kcal/mol with DRD1, binding within its hydrophobic pocket, and a score of –6.9 kcal/mol with GRK4, also binding within its hydrophobic pocket. These findings indicate that salidroside has a strong binding affinity for both DRD1 and GRK4, suggesting it may regulate dopamine signaling by directly targeting these proteins. The potential effects of salidroside binding to DRD1 and GRK4 may differ. Binding to DRD1 could help maintain the receptor in an active state and support dopamine-mediated signaling under hypoxic conditions, while binding to GRK4 might reduce its kinase activity, thereby preventing excessive DRD1 phosphorylation and desensitization. These structural predictions are consistent with previous studies showing that GRK4 negatively regulates DRD1 function through hyperphosphorylation and internalization. However, molecular docking alone does not explain the observed changes in DRD1 and GRK4 mRNA expression. Such transcriptional alterations are more likely secondary to salidroside’s broader regulatory effects—such as its antioxidant and anti-inflammatory actions—rather than a direct result of receptor binding. To further validate this hypothesis, we conducted animal experiments involving salidroside intervention under hypobaric hypoxia. In rats treated with various doses of salidroside, GRK4 expression in renal tissue was decreased on days 7, 14, and 28 compared to corresponding time points in the untreated hypoxia group. Conversely, DRD1 protein expression was increased on days 3, 7, 14, and 28. These results suggest that salidroside may reduce GRK4 expression in the kidney, thereby weakening the inhibitory effect of GRK4 on DRD1, and enhancing DRD1 function to some extent. This modulation may represent one of the potential mechanisms by which salidroside confers renoprotective effects under simulated high-altitude conditions. Interestingly, salidroside exhibited a time-dependent effect on renal injury biomarkers, showing significant attenuation of NGAL, KIM-1, and Cys-C at early time points (days 3 and 7), followed by a transient rebound at day 14 and stabilization by day 28. However, the temporal variation of DRD1 and GRK4 expression was not completely consistent with these changes. This difference may reflect distinct physiological dynamics between acute tubular injury markers and receptor-mediated signaling regulation. While NGAL, KIM-1, and Cys-C are sensitive indicators of early kidney stress and recovery, alterations in DRD1 and GRK4 expression likely represent more sustained molecular adaptations to hypoxic exposure and salidroside treatment.

We further investigated whether the renal protective effect of salidroside is associated with the regulation of GRK4 or DRD1 expression through in vitro experiments. Specifically, we used the dopamine receptor agonist fenoldopam and RNAi-mediated DRD1 silencing to evaluate RPT cell injury under different conditions. Under normoxic conditions, RPT cells exhibited low GRK4 protein expression and high DRD1 protein expression. In contrast, after 24 hours of hypoxic culture, the levels of NGAL and KIM-1 in the culture supernatant were significantly increased. At the same time, GRK4 expression was markedly upregulated, while DRD1 expression was significantly downregulated. Following salidroside treatment, the levels of NGAL and KIM-1 were reduced and the extent of cell apoptosis was alleviated compared with the hypoxia group. Correspondingly, GRK4 expression decreased, and DRD1 expression increased, consistent with the observed reduction in cell injury. Similar to the results observed in animal experiments, these findings suggest that salidroside may exert its protective effects by modulating GRK4 or DRD1 expression. Further intervention experiments confirmed that fenoldopam could attenuate cell apoptosis. In contrast, silencing DRD1 weakened the protective effect of salidroside on RPT cells, as evidenced by elevated levels of NGAL and KIM-1 and increased apoptosis. Interestingly, salidroside showed a stronger inhibitory effect on GRK4 expression under hypoxic conditions than under normoxia. This may be because hypoxia activates oxidative and inflammatory pathways that enhance GRK4 expression, making the suppressive effect of salidroside more evident. Although GRK4 is generally considered an upstream regulator of DRD1 phosphorylation and desensitization, recent studies suggest possible feedback regulation between the two proteins. Reduced DRD1 signaling may influence GRK4 expression through downstream pathways such as PKA or MAPK, resulting in compensatory downregulation of GRK4 in siDRD1 cells. Collectively, these results further confirm the protective role of salidroside in RPT cells under hypoxic conditions, which is likely associated with the regulation of GRK4 and DRD1 expression.

However, the hypobaric hypoxia chamber used in this study does not fully replicate the actual high-altitude environment. In addition, we only examined changes in GRK4 and DRD1 expression in rat kidneys. The lack of GRK4 knockdown and DRD1 transgenic animal models limits our ability to validate the proposed molecular mechanisms, which represents a limitation of the present study. Moreover, systemic blood pressure and renal hemodynamic parameters (such as renal blood flow and glomerular filtration rate) were not assessed in this study. Given that the renal dopaminergic system is known to influence sodium excretion and blood pressure regulation, future studies will include these measurements to better clarify the physiological impact of salidroside on cardiovascular–renal function under hypoxic conditions. Furthermore, although the molecular docking analysis was performed using AutoDock Vina, which provides reliable predictions of ligand-receptor interactions, validation using more advanced AI-based docking platforms (such as DeepDock) or experimental binding assays was not conducted. Future studies incorporating these approaches would further clarify the precise binding mechanisms of salidroside with DRD1 and GRK4 and enhance the mechanistic robustness of our findings.

In summary, our study revealed that a hypobaric hypoxic environment simulating an altitude of 5000 meters induces renal injury in rats. Salidroside was found to alleviate such injury, showing potential as a protective agent for the prevention of acute high-altitude renal damage. This protective effect may be associated with the inhibition of local renal GRK4 expression and the restoration of DRD1 expression.

## Supporting information

S1 Table Raw data underlying Fig 3. Original quantitative data for serum and urinary KIM-1, NGAL, and Cys-C measurements corresponding to Fig 3. (XLSX)

S2 Table Raw data underlying Fig 11. Original quantitative data for NGAL and KIM-1 levels measured in RPT cell culture supernatants corresponding to Fig 11. (XLSX)

S1 Fig Uncropped Western blot images for Fig 5. Original, uncropped Western blot images showing GRK4 and DRD1 protein expression in rat kidney tissues after hypoxia exposure. (PDF)

S2 Fig Uncropped Western blot images for Fig 6. Original, uncropped Western blot images showing GRK4 and DRD1 protein expression in rat kidney tissues following salidroside treatment at different time points. (PDF)

S3 Fig Uncropped Western blot images for Fig 14. Original, uncropped Western blot images showing GRK4 and DRD1 protein expression in RPT cells under different experimental conditions. (PDF)
