# Correction: Salidroside protects against high-altitude hypoxia-induced kidney injury via regulation of renal dopamine D1-like receptors

**DOI:** 10.1371/journal.pone.0351250

**Published:** 2026-06-08

**Authors:** Cheng Huan, Gan Zhilin, Xiao Dan, Wang Yue, Li Xianglian, Mo Liwen, Cheng Yue

There are errors in the author affiliations. The correct affiliations are as follows:

Cheng Huan^1,2^, Gan Zhilin^1^, Xiao Dan^1^, Wang Yue^1^, Li Xianglian^1^, Mo Liwen^1^, Cheng Yue^1,2^

**1** Department of Nephrology, General Hospital of Western Theater Command of PLA, Chengdu, PR China, **2** College of Medicine, Southwest Jiaotong University, Chengdu, PR China
