# Supplementary material for: Salidroside protects against high-altitude hypoxia-induced kidney injury via regulation of renal dopamine D1-like receptors
Source: PLoS One. 2026 Mar 31;21(3):e0344999. doi: 10.1371/journal.pone.0344999 (PMC13037985; doi:10.1371/journal.pone.0344999)
Supplement: S1 Fig — (PDF) [file pone.0344999.s003.pdf]

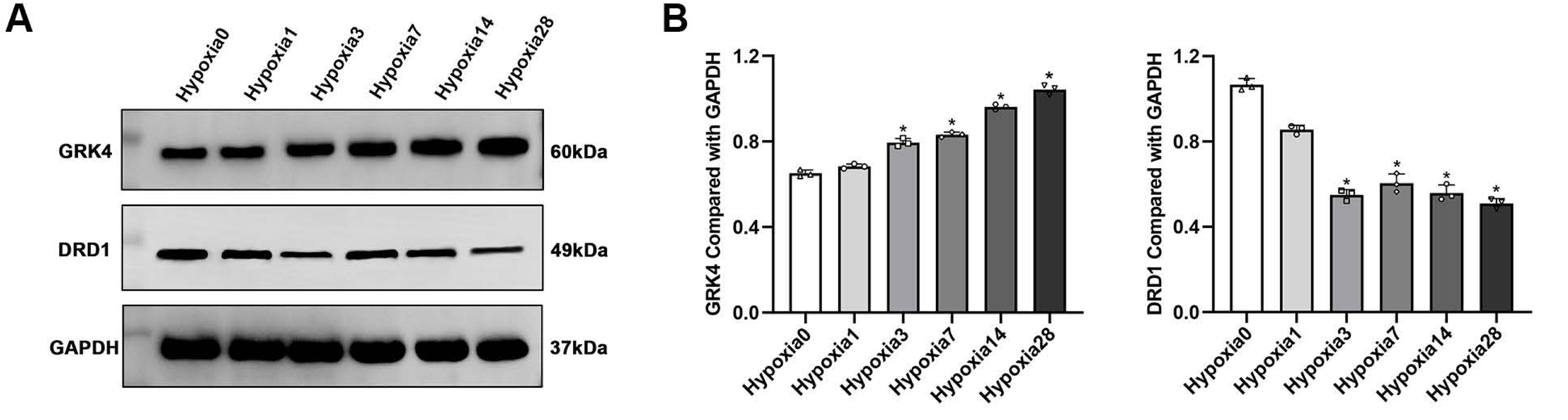

A
Hypoxia0
Hypoxia1
Hypoxia3
Hypoxia7
Hypoxia14
Hypoxia28
GRK4
60kDa
DRD1
49kDa
GAPDH
37kDa
B
GRK4 Compared with GAPDH
1.2
0.8
0.4
0.0
Hypoxia0
Hypoxia1
Hypoxia3
Hypoxia7
Hypoxia14
Hypoxia28
DRD1 Compared with GAPDH
1.2
0.8
0.4
0.0
Hypoxia0
Hypoxia1
Hypoxia3
Hypoxia7
Hypoxia14
Hypoxia28

Fig5-DRD1(49kDa)

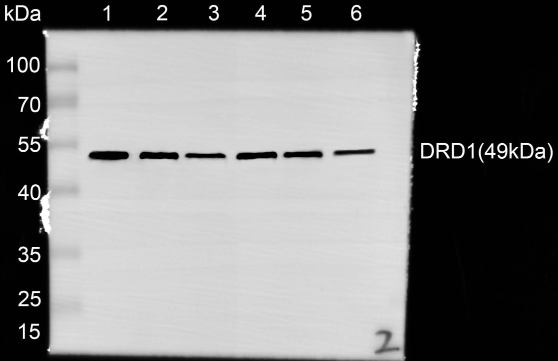

Lanes:
1.Hypoxia0,the concentraion is 25μg/ml.
2.Hypoxia1,the concentraion is 25μg/ml.
3.Hypoxia3,the concentraion is 25μg/ml
4.Hypoxia7,the concentraion is 25μg/ml.
5.Hypoxia14,the concentraion is 25μg/ml.
6.Hypoxia28,the concentraion is 25μg/ml..

Fig5-GAPDH(37kDa)

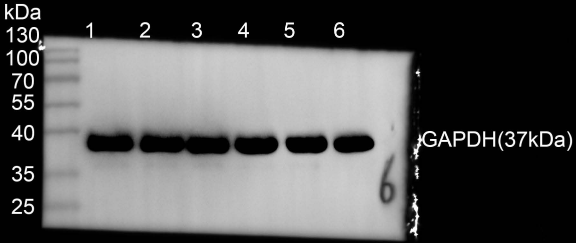

Lanes:

1.Hypoxia0,the concentraion is 25μg/ml.
2.Hypoxia1,the concentraion is 25μg/ml.
3.Hypoxia3,the concentraion is 25μg/ml
4.Hypoxia7,the concentraion is 25μg/ml.
5.Hypoxia14,the concentraion is 25μg/ml.
6.Hypoxia28,the concentraion is 25μg/ml..

Fig5-GRK4(60kDa)

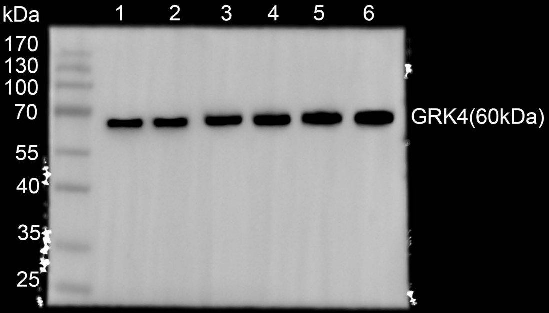

Lanes:

1.Hypoxia0,the concentraion is 25μg/ml.
2.Hypoxia1,the concentraion is 25μg/ml.
3.Hypoxia3,the concentraion is 25μg/ml
4.Hypoxia7,the concentraion is 25μg/ml.
5.Hypoxia14,the concentraion is 25μg/ml.
6.Hypoxia28,the concentraion is 25μg/ml..
