# Supplementary material for: Salidroside protects against high-altitude hypoxia-induced kidney injury via regulation of renal dopamine D1-like receptors
Source: PLoS One. 2026 Mar 31;21(3):e0344999. doi: 10.1371/journal.pone.0344999 (PMC13037985; doi:10.1371/journal.pone.0344999)
Supplement: S2 Fig — (PDF) [file pone.0344999.s004.pdf]

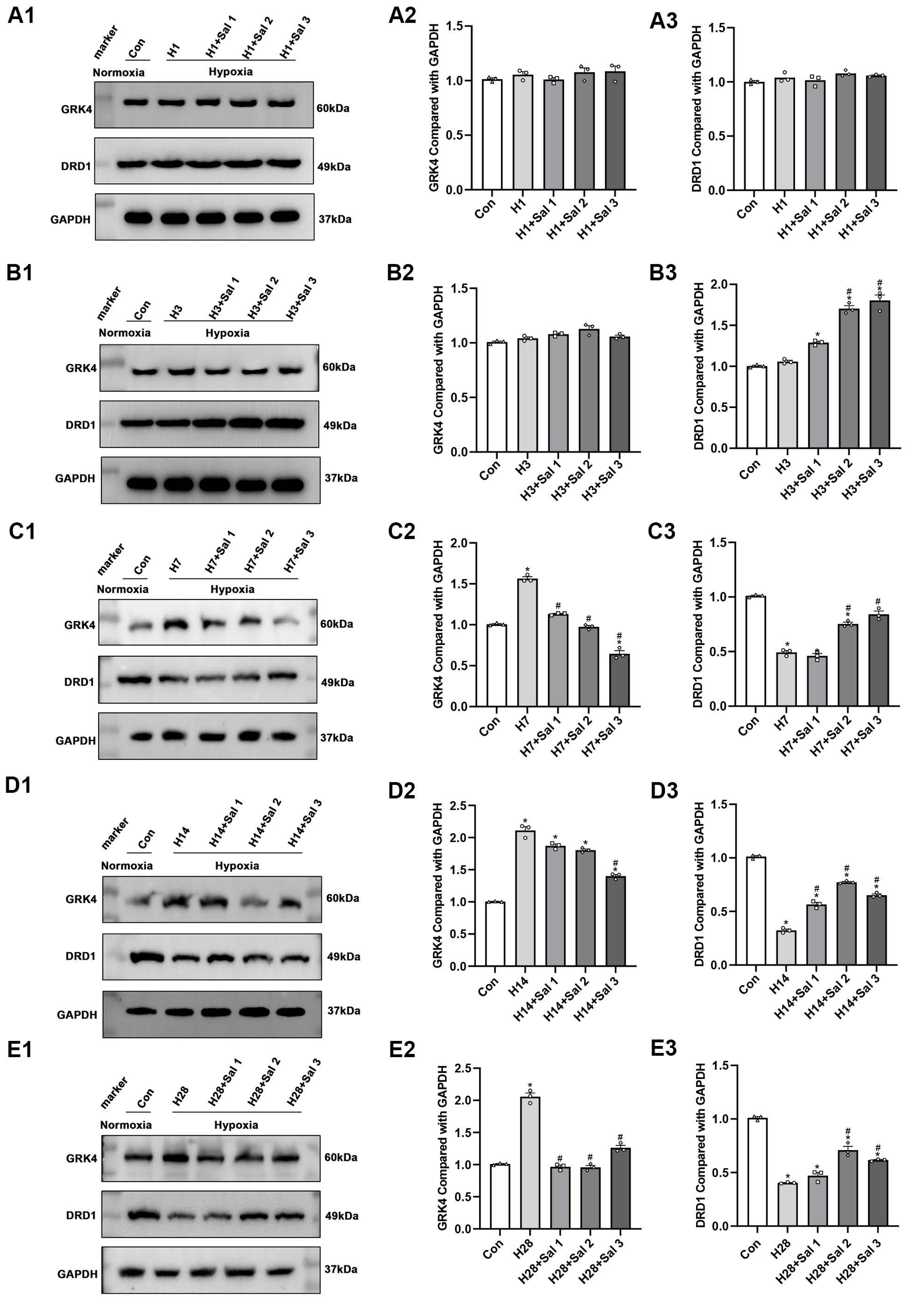
A1
marker
Con
H1
H1+Sal 1
H1+Sal 2
H1+Sal 3
Normoxia
Hypoxia
GRK4
DRD1
GAPDH
60kDa
49kDa
37kDa
A2
GRK4 Compared with GAPDH
A3
DRD1 Compared with GAPDH
B1
H3
H3+Sal 1
H3+Sal 2
H3+Sal 3
B2
B3
C1
H7
H7+Sal 1
H7+Sal 2
H7+Sal 3
C2
C3
D1
H14
H14+Sal 1
H14+Sal 2
H14+Sal 3
D2
D3
E1
H28
H28+Sal 1
H28+Sal 2
H28+Sal 3
E2
E3

Fig6 A1-DRD1(49kDa)

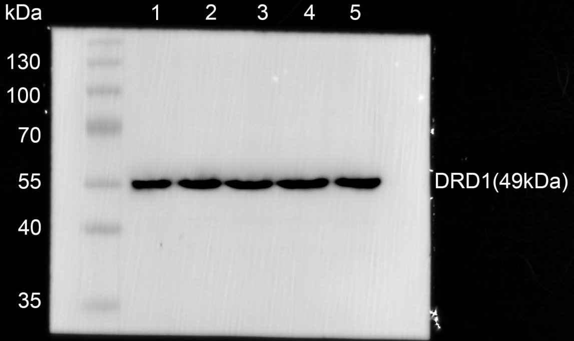

Lanes:
1.Con,the concentraion is 30μg/ml.
2.H1,the concentraion is 30μg/ml.
3.H1+20mg/kg Sal1,the concentraion is 30μg/ml
4.H1+40mg/kg Sal2,the concentraion is 30μg/ml.
5.H1+60mg/kg Sal3,the concentraion is 30μg/ml.

Fig6 A1-GAPDH(37kDa)

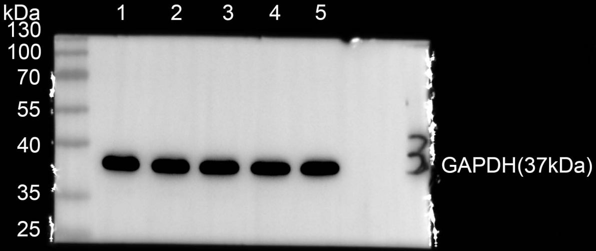

Lanes:
1.Con,the concentraion is 30mg/ml.
2.H1,the concentraion is 30mg/ml.
3.H1+20mg/kg Sal1,the concentraion is 30mg/ml
4.H1+40mg/kg Sal2,the concentraion is 30mg/ml.
5.H1+60mg/kg Sal3,the concentraion is 30mg/ml.

Fig6 A1-GRK4(60kDa)

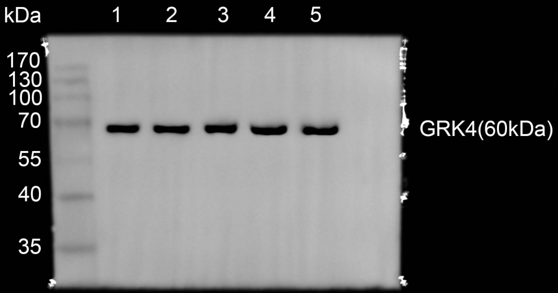

Lanes:

1.Con,the concentraion is 30μg/ml.

2.H1,the concentraion is 30μg/ml.

3.H1+20mg/kg Sal1,the concentraion is 30μg/ml

4.H1+40mg/kg Sal2,the concentraion is 30μg/ml.

5.H1+60mg/kg Sal3,the concentraion is 30μg/ml.

Fig6 B1-DRD1(49kDa)

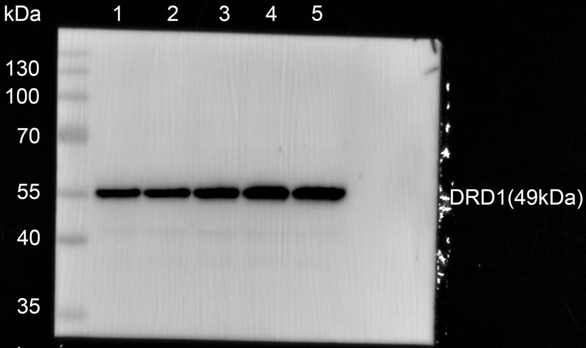

Lanes:

1.Con,the concentraion is 30μg/ml.

2.H3,the concentraion is 30μg/ml.

3.H3+20mg/kg Sal1,the concentraion is 30μg/ml

4.H3+40mg/kg Sal2,the concentraion is 30μg/ml.

5.H3+60mg/kg Sal3,the concentraion is 30μg/ml.

Fig6 B1-GAPDH(37kDa)

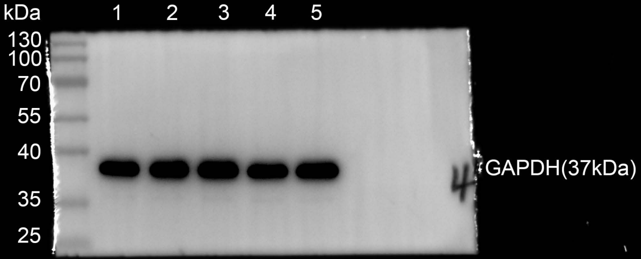

Lanes:

1.Con,the concentraion is 30μg/ml.

2.H3,the concentraion is 30μg/ml.

3.H3+20mg/kg Sal1,the concentraion is 30μg/ml

4.H3+40mg/kg Sal2,the concentraion is 30μg/ml.

5.H3+60mg/kg Sal3,the concentraion is 30μg/ml.

Fig6 B1-GRK4(60kDa)

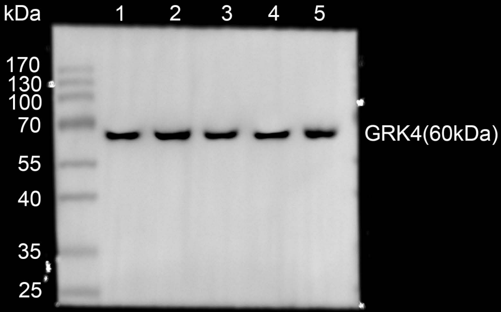

Lanes:

1.Con,the concentraion is 30μg/ml.

2.H3,the concentraion is 30μg/ml.

3.H3+20mg/kg Sal1,the concentraion is 30μg/ml

4.H3+40mg/kg Sal2,the concentraion is 30μg/ml.

5.H3+60mg/kg Sal3,the concentraion is 30μg/ml.

Fig6 C1-DRD1(49kDa)

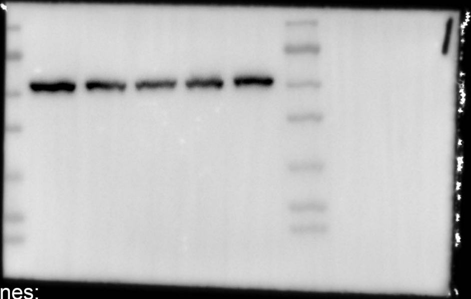

Lanes:
1.Con,the concentraion is 30μg/ml.
2.H7,the concentraion is 30μg/ml.
3.H7+20mg/kg Sal1,the concentraion is 30μg/ml
4.H7+40mg/kg Sal2,the concentraion is 30μg/ml.
5.H7+60mg/kg Sal3,the concentraion is 30μg/ml.

Fig6 C1-GAPDH(37kDa)

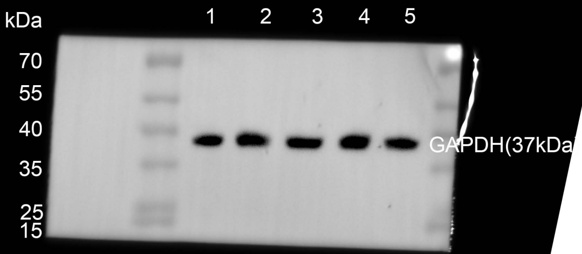

Lanes:
1.Con,the concentraion is 30μg/ml.
2.H7,the concentraion is 30μg/ml.
3.H7+20mg/kg Sal1,the concentraion is 30μg/ml
4.H7+40mg/kg Sal2,the concentraion is 30μg/ml.
5.H7+60mg/kg Sal3,the concentraion is 30μg/ml.

Fig6 C1-GRK4(60kDa)

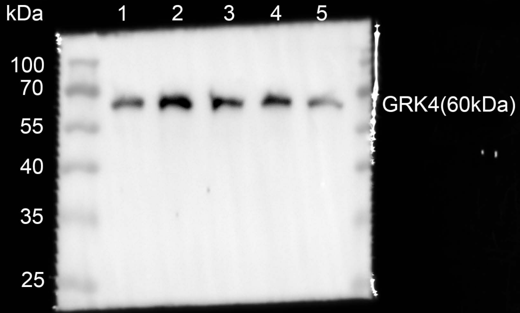

Lanes:
1.Con,the concentraion is 30μg/ml.
2.H7,the concentraion is 30μg/ml.
3.H7+20mg/kg Sal1,the concentraion is 30μg/ml
4.H7+40mg/kg Sal2,the concentraion is 30μg/ml.
5.H7+60mg/kg Sal3,the concentraion is 30μg/ml.

Fig6 D1-DRD1(49kDa)

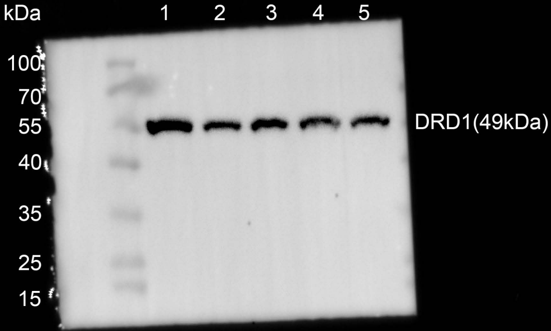

Lanes:
1.Con,the concentraion is 30μg/ml.
2.H14,the concentraion is 30μg/ml.
3.H14+20mg/kg Sal1,the concentraion is 30μg/ml
4.H14+40mg/kg Sal2,the concentraion is 30μg/ml.
5.H14+60mg/kg Sal3,the concentraion is 30μg/ml.

Fig6 D1-GAPDH(37kDa)

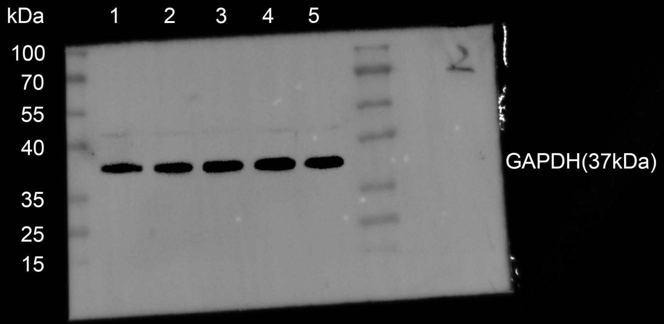

Lanes:

1.Con,the concentraion is 30μg/ml.

2.H14,the concentraion is 30μg/ml.

3.H14+20mg/kg Sal1,the concentraion is 30μg/ml

4.H14+40mg/kg Sal2,the concentraion is 30μg/ml.

5.H14+60mg/kg Sal3,the concentraion is 30μg/ml.

Fig6 D1-GRK4(60kDa)

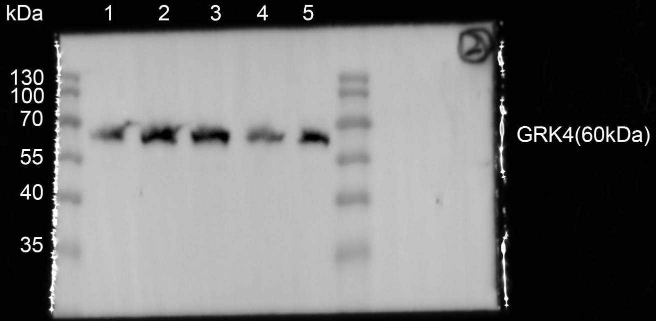

Lanes:

1.Con,the concentraion is 30μg/ml.

2.H14,the concentraion is 30μg/ml.

3.H14+20mg/kg Sal1,the concentraion is 30μg/ml

4.H14+40mg/kg Sal2,the concentraion is 30μg/ml.

5.H14+60mg/kg Sal3,the concentraion is 30μg/ml.

Fig6 E1-DRD1(49kDa)

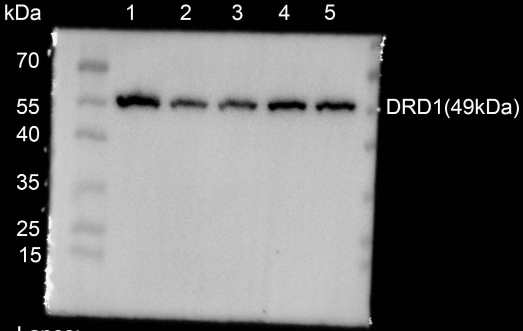

Lanes:

1.Con,the concentraion is 30μg/ml.
2.H28,the concentraion is 30μg/ml.
3.H28+20mg/kg Sal1,the concentraion is 30μg/ml
4.H28+40mg/kg Sal2,the concentraion is 30μg/ml.
5.H28+60mg/kg Sal3,the concentraion is 30μg/ml.

Fig6 E1-GAPDH(37kDa)

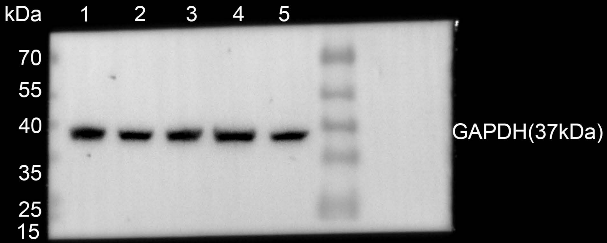

Lanes:

1.Con,the concentraion is 30μg/ml.

2.H28,the concentraion is 30μg/ml.

3.H28+20mg/kg Sal1,the concentraion is 30μg/ml

4.H28+40mg/kg Sal2,the concentraion is 30μg/ml.

5.H28+60mg/kg Sal3,the concentraion is 30μg/ml.

Fig6 E1-GRK4(60kDa)

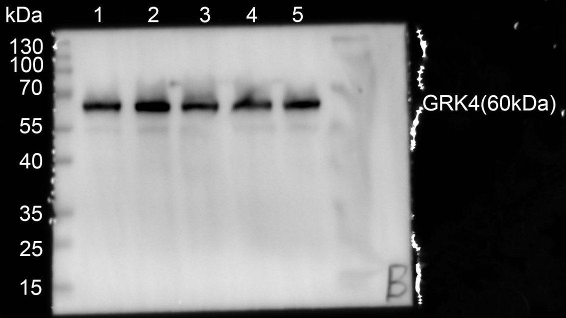

Lanes:

1.Con,the concentraion is 30μg/ml.

2.H28,the concentraion is 30μg/ml.

3.H28+20mg/kg Sal1,the concentraion is 30μg/ml

4.H28+40mg/kg Sal2,the concentraion is 30μg/ml.

5.H28+60mg/kg Sal3,the concentraion is 30μg/ml.
