# Supplementary material for: Salidroside protects against high-altitude hypoxia-induced kidney injury via regulation of renal dopamine D1-like receptors
Source: PLoS One. 2026 Mar 31;21(3):e0344999. doi: 10.1371/journal.pone.0344999 (PMC13037985; doi:10.1371/journal.pone.0344999)
Supplement: S3 Fig — (PDF) [file pone.0344999.s005.pdf]

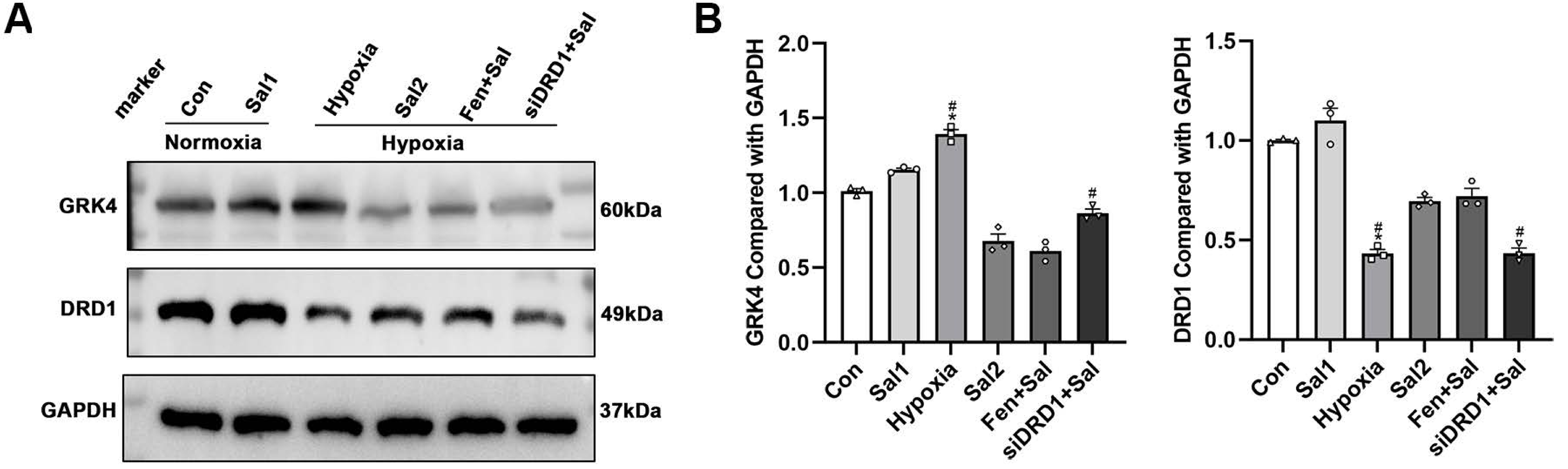
A
marker
Con
Sal1
Hypoxia
Sal2
Fen+Sal
siDRD1+Sal
Normoxia
Hypoxia
GRK4
60kDa
DRD1
49kDa
GAPDH
37kDa
B
GRK4 Compared with GAPDH
2.0
1.5
1.0
0.5
0.0
Con
Sal1
Hypoxia
Sal2
Fen+Sal
siDRD1+Sal
DRD1 Compared with GAPDH
1.5
1.0
0.5
0.0
Con
Sal1
Hypoxia
Sal2
Fen+Sal
siDRD1+Sal

Fig14 A-DRD1(49kDa)

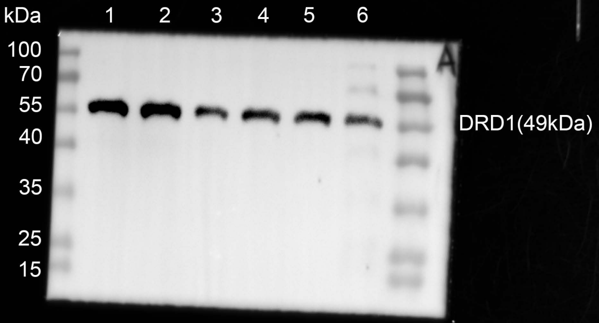

Lanes:

1.Con,the concentraion is 25μg/ml.

2.25μM Sal1,the concentraion is 25μg/ml

3.Hypoxia,the concentraion is 25μg/ml

4.Hypoxia+25μM Sal2,the concentraion is 25μg/ml

5.Hypoxia+1μM Fen+25μM Sal2,the concentraion is 25μg/ml

6.Hypoxia+siDRD1+25μM Sal2,the concentraion is 25μg/ml

Fig14 A-GAPDH(37kDa)

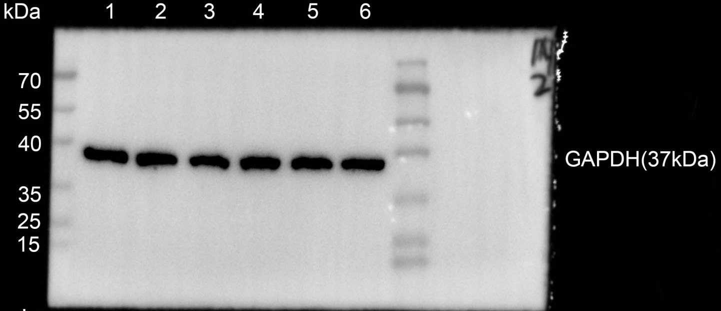

Lanes:

1.Con,the concentraion is 25μg/ml.

2.25μM Sal1,the concentraion is 25μg/ml

3.Hypoxia,the concentraion is 25μg/ml

4.Hypoxia+25μM Sal2,the concentraion is 25μg/ml

5.Hypoxia+1μM Fen+25μM Sal2,the concentraion is 25μg/ml

6.Hypoxia+siDRD1+25μM Sal2,the concentraion is 25μg/ml

Fig14 A-GRK4(60kDa)

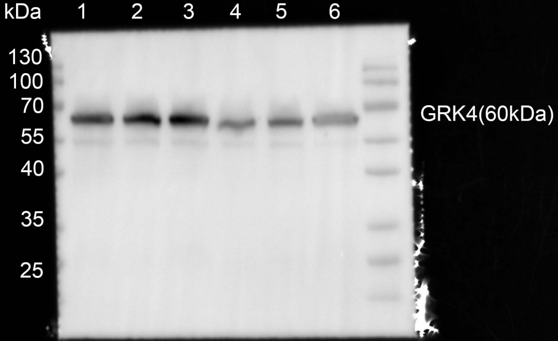

Lanes:
1.Con,the concentraion is 25μg/ml.
2.25μM Sal1,the concentraion is 25μg/ml
3.Hypoxia,the concentraion is 25μg/ml
4.Hypoxia+25μM Sal2,the concentraion is 25μg/ml
5.Hypoxia+1μM Fen+25μM Sal2,the concentraion is 25μg/ml
6.Hypoxia+siDRD1+25μM Sal2,the concentraion is 25μg/ml
